# Deodeokaloid, a New Indole Alkaloid *N*-Glycoside and Bioactive Phenolic Compounds from the Roots of *Codonopsis lanceolata*

**DOI:** 10.3390/plants13223243

**Published:** 2024-11-19

**Authors:** Yeo Rang Cho, Joo-Hyun Hong, Dong-Min Kang, Yoon-Joo Ko, Mi-Jeong Ahn, Ki Hyun Kim

**Affiliations:** 1 School of Pharmacy, Sungkyunkwan University, Suwon 16419, Republic of Korea; yr6755@g.skku.edu (Y.R.C.); ehong@skku.edu (J.-H.H.); 2 College of Pharmacy and Research Institute of Pharmaceutical Sciences, Gyeongsang National University, Jinju 52828, Republic of Korea; kdm7105@gnu.ac.kr; 3 Laboratory of Nuclear Magnetic Resonance, National Center for Inter-University Research Facilities (NCIRF), Seoul National University, Seoul 08826, Republic of Korea; yjko@snu.ac.kr

**Keywords:** *Codonopsis lanceolata*, indole alkaloid glycoside, *Helicobacter pylori*, nitric oxide, antioxidant activity

## Abstract

*Codonopsis lanceolata*, commonly known as the bonnet bellflower or deodeok, is primarily found in Eastern Asia. Its roots have been used traditionally across Asia to treat various ailments such as bronchitis, coughs, asthma, and inflammation. In our ongoing efforts to discover bioactive natural products, a phytochemical investigation of the *n*-BuOH fraction of *C. lanceolata* root extracts led to the isolation and identification of a new indole alkaloid *N*-glycoside, deodeokaloid (D-indole-3-lactic acid *N*-β-D-glucopyranoside) (**1**), alongside known compounds tangshenoside I (**2**), tangshenoside IV (**3**), and chlorogenic acid (**4**) through HPLC purification. The structure of the new compound **1** was elucidated using 1D and 2D NMR spectroscopy and high-resolution electrospray ionization mass spectrometry (HR-ESIMS). Its absolute configuration was determined through a combination of DP4+ probability analysis and chemical reactions. The isolated compounds **1**–**4** were evaluated for their anti-*Helicobacter pylori* and antioxidant activities. In the anti-*H. pylori* assay, compound **3** showed antibacterial activity similar to that of quercetin as the positive control, inhibiting the bacterial growth by 36.8%. Compound **4** exhibited the most potent antioxidant activity, with an ABTS [2,2′-azino-bis(3-ethylbenzothiazoline-6-sulfonate)] radical scavenging activity of 1624.7 mmol TE/mol and a DPPH (2,2-diphenyl-1-picrylhydrazyl) radical scavenging capacity of 707.5 mmol Trolox equivalent (TE)/mol. Compounds **2**–**4** displayed significant intracellular reactive oxygen species (ROS) scavenging capacity in lipopolysaccharide-stimulated RAW 264.7 macrophage cells. This study highlights *C. lanceolata* roots as a promising natural source of bioactive compounds with potential therapeutic applications.

## 1. Introduction

The perennial herb *Codonopsis lanceolata*, commonly known as bonnet bellflower, belongs to the Campanulaceae family. It is widely distributed throughout Korea, China, and Japan and is one of the most commonly cultivated vegetables in Korea. The roots of *C. lanceolata* are consumed both raw and cooked and have a long-standing reputation in traditional East Asian medicine for treating various inflammatory disorders such as bronchial asthma, coughs, tonsillitis, and pharyngitis [1,2]. Known locally as deodeok in Korea, the roots of *C. lanceolata* are widely consumed as a food and used in traditional herbal medicine, valued for their therapeutic properties in treating bronchitis, cough, asthma, and inflammation [3,4].

Recent studies have demonstrated the broad therapeutic potential of *C. lanceolata* extract. It has been reported to protect against liver damage [5], combat obesity [6], enhance memory development [7], and inhibit tumor growth [8]. Specifically, the extract has shown effectiveness in preventing hypertension and reducing systolic blood pressure (SBP) in hypertensive rats at doses of 200 mg and 400 mg per kg body weight, without affecting SBP in normotensive rats [9]. Phytochemical investigations have identified a diverse array of secondary metabolites in the roots of *C. lanceolata*, including saponins [10], triterpenoids [11], phenylpropanoids [12], and alkaloids [13]. These compounds exhibit a wide range of pharmacological activities, such as anti-inflammatory, anti-stress, antioxidant, anticancer, and immunomodulatory effects [14,15]. Significantly, research has highlighted that chronic inflammatory conditions caused by *Helicobacter pylori* infection or oxidative stress can lead to severe diseases, including gastric cancer [16]. This has spurred interest in the roots of *C. lanceolata* for their anti-inflammatory and antioxidant properties. Notably, lancemaside A, a major triterpenoid saponin identified in *C. lanceolata* roots, has been shown to inhibit IκB kinase(IKK)/nuclear factor-κappa B (NF-κB) activation in RAW 264.7 and U937 cells, significantly enhancing anti-inflammatory responses [17]. Moreover, it effectively alleviates colitis through TLR-linked NF-κB activation in murine models [18]. Despite these insights, research on *C. lanceolata* has predominantly focused on lancemaside A and its triterpenoid saponins. Consequently, there is a pressing need to explore *C. lanceolata* further to identify novel bioactive compounds with varied structural features. Phytochemical studies on other species within the *Codonopsis* genus, such as *C. clematidea* and *C. ovata*, have identified unique bioactive constituents. For instance, these species contain pyrrolidine-type alkaloids including codonopsine and codonopsinine [19], which have demonstrated blood-pressure-lowering effects in cats at doses of 20 mg/kg without affecting the central nervous system. Additionally, phenylpropanoids, lignans, and flavonoids isolated from the roots of *C. cordifolioidea* have shown promising anti-HIV-1 activity with an EC_50_ value of 2.3 µg/mL and exhibit cytotoxic effects against various tumor cell lines including HL-60, Hep-G2, KB, and MDA-MB-231 [20]. These findings underscore the potential of *Codonopsis* species as a source of therapeutically bioactive natural compounds. 

As part of a continuing exploration of novel and bioactive metabolites from intriguing natural sources [21,22,23,24,25], we initiated a study focusing on identifying potential new bioactive compounds within the ethanol (EtOH) extract of *C. lanceolata* roots since the EtOH extract demonstrated anti-*Helicobacter pylori* activity in preliminary bioactive screening tests. In this study, the EtOH extract of *C. lanceolata* roots underwent solvent partitioning, with subsequent LC/MS analysis revealing that the *n*-BuOH fraction contained key constituents such as alkaloids, phenolic glycosides, and phenylpropanoids. Comprehensive chemical analysis of this fraction through repeated column chromatography and semi-preparative HPLC resulted in the isolation and identification of four compounds (**1**–**4**), including a new indole alkaloid *N*-glycoside (**1**) and three known phenolic compounds (**2**–**4**). The structure of the new compound (**1**) was established through detailed 1D and 2D NMR spectroscopic techniques (^1^H-^1^H COSY, HSQC, and HMBC), high-resolution electrospray ionization mass spectrometry (HR-ESIMS), and chemical reactions. Furthermore, our study investigated the potential bioactivities of these compounds, particularly focusing on their anti-*Helicobacter pylori* and antioxidant activities.

## 2. Results and Discussion

### 2.1. Isolation and Structural Elucidation of Compounds ***1**–**4***

**Figure 1:**
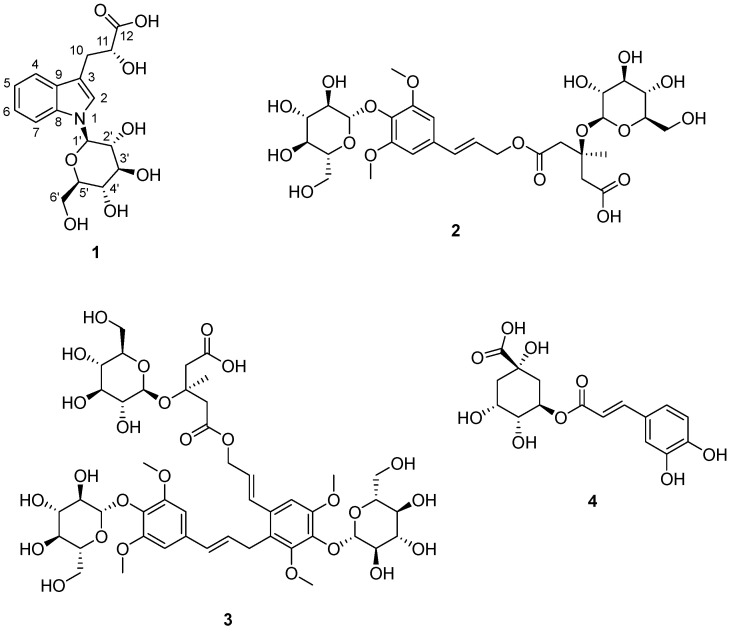
Chemical structures of compounds **1**–**4**.

The root of *C. lanceolata* was extracted with 50% ethanol, resulting in an ethanol (EtOH) extract. Preliminary bioactive screening tests revealed that the EtOH extract exhibited anti-*Helicobacter pylori* activity, showing 12.6 ± 1.9% inhibition at a concentration of 250 μg/mL. To identify potential bioactive compounds, the EtOH extract was fractionated by solvent partitioning into *n*-hexane-, dichloromethane (CH_2_Cl_2_)-, ethyl acetate (EtOAc)-, and *n*-butanol (*n*-BuOH)-soluble fractions. The *n*-BuOH fraction was selected for further study due to its rich content of significant compounds, including alkaloids, phenolic glycosides such as tangshenosides, and phenylpropanoids, by analysis of LC/MS and HR-LC/MS data. The *n*-BuOH fraction underwent extensive phytochemical investigation using repeated column chromatography and both preparative and semi-preparative high-performance liquid chromatography (HPLC). These purification steps led to the isolation of a new indole alkaloid *N*-glycoside (**1**) and three known phenolic compounds (**2**–**4**), as illustrated in Figure 1.

Compound **1** was isolated as a colorless gum. The molecular formula of **1** was assigned as C_17_H_21_NO_8_, determined by the positive-ion mode of HR-ESIMS, which demonstrated an [M + H]^+^ ion peak at *m*/*z* 368.1351 (calcd. for C_17_H_22_NO_8_, 368.1345). The ^1^H NMR data (Table 1) of **1**, assigned with the aid of a heteronuclear single quantum correlation (HSQC) experiment, displayed characteristic proton signals of an indole ring group at *δ*_H_ 7.59 (1H, d, *J* = 8.0 Hz, H-4), 7.50 (1H, d, *J* = 8.0 Hz, H-7), 7.31 (1H, s, H-2), 7.15 (1H, t, *J* = 8.0 Hz, H-6), and 7.06 (1H, t, *J* = 8.0 Hz, H-5), together with one oxygenated methine proton at *δ*_H_ 4.41 (1H, br s, H-11) and one methylene group at *δ*_H_ 3.24 (1H, d, *J* = 14.0 Hz, H-10a) and 3.09 (1H, dd, *J* = 14.0, 7.0 Hz, H-10b). In addition, a characteristic anomeric proton signal was observed at *δ*_H_ 5.42 (1H, d, *J* = 9.0 Hz, H-1′), which indicates the presence of a sugar moiety in the compound **1**. The ^13^C NMR data (Table 1) of **1**, in combination with HSQC and heteronuclear multiple bond correlation (HMBC) analysis, revealed 17 carbon resonances including three non-protonated carbons [*δ*_C_ 138.3, 130.2, and 112.7], four protonated aromatic carbons [*δ*_C_ 122.8, 120.7, 119.9, and 111.4], one olefinic carbon [*δ*_C_ 125.4], an oxygenated methine group [*δ*_C_ 71.9], a methylene group [*δ*_C_ 31.4], and a carbonyl group [*δ*_C_ 179.1], as well as *N*-glucoside carbon signals [*δ*_C_ 86.7, 80.5, 79.0, 73.7, 71.5, and 62.8] [26]. The observed ^1^H and ^13^C NMR spectroscopic features suggested that compound **1** may be an indole alkaloid *N*-glycoside that is similar to indole-3-lactic acid glycoside [27], except for the ^1^H and ^13^C NMR chemical shifts of anomeric position and the coupling constant of the anomeric proton (H-1′). In previous studies, the anomeric proton of indole-3-lactic acid *β*-D-glucoside was observed in the ^1^H NMR data at *δ*_H_ 4.35 (1H, d, *J* = 7.4 Hz) and 4.34 (1H, d, *J* = 7.3 Hz), respectively [27]. However, in these data, it was observed at 5.42 (1H, d, *J* = 9.0 Hz), suggesting that the sugar moiety is attached at a different position compared to the previously isolated compound, indole-3-lactic acid glycoside. The position of the sugar moiety in compound **1** was determined to be *N*-glucoside, based on the chemical shift and coupling constant of the anomeric proton in tryptophan-*N*-glucoside [26].

**Figure 2:**
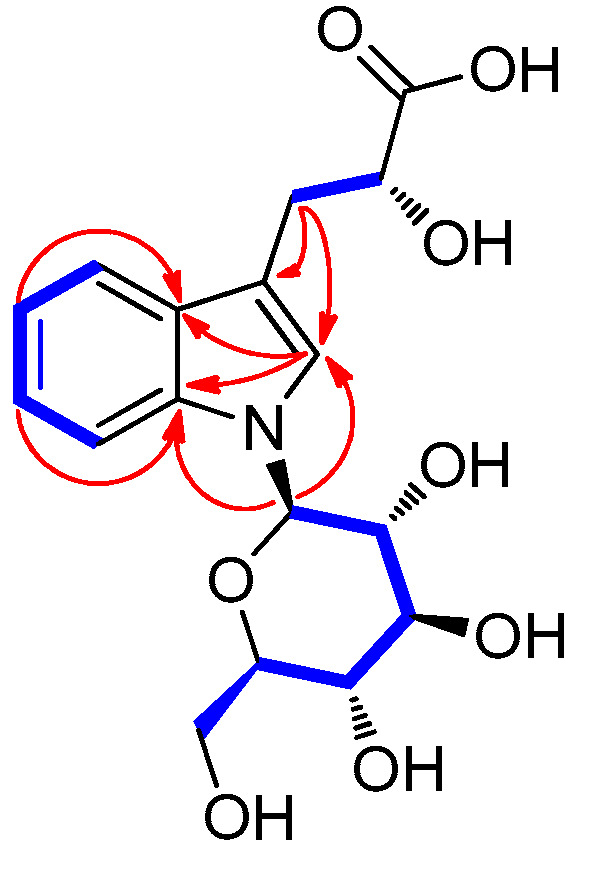
Key ^1^H-^1^H COSY (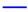) and HMBC (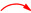) correlations for compound **1**.

The planar structure of **1** was elucidated by the analysis of 2D NMR (COSY and HMBC) experiments (Figure 2). The ^1^H-^1^H COSY correlations of H-4/H-5/H-6/H-7, H-10/H-11, and H-1′/H-2′/H-3′/H-4′/H-5′/H-6′ were observed. The HMBC correlations from H-2 to C-8; from H-2 to C-9; from H-5 to C-9; from H-6 to C-8; from H-10 to C-2; and from H-10 to C-3 indicated the presence of indole-3-lactic acid (Figure 2). The key HMBC correlations of H-1′/C-2 and H-1′/C-8 confirmed the connection of glucoside at the *N*-atom of indole-3-lactic acid (Figure 2). To determine the absolute configuration of the sugar unit in compound **1**, enzymatic hydrolysis was performed using β-glucosidase (8.0 mg, from almonds, Sigma-Aldrich) [28], which confirmed the D-configuration. This conclusion was established by comparing the retention time of derivatives for the enzymatic hydrolysate of compound **1** (*t*_R_ 5.9 min) with those of standard samples of D-glucopyranose (*t*_R_ 6.2 min) and L-glucopyranose (*t*_R_ 1.7 min) in LC/MS analysis. Furthermore, the coupling constant (*J* = 9.0 Hz) of the anomeric proton signal confirmed the β-form of glucopyranose, indicating the sugar in compound **1** was *β*-D-glucopyranose [26,29].

**Figure 3:**
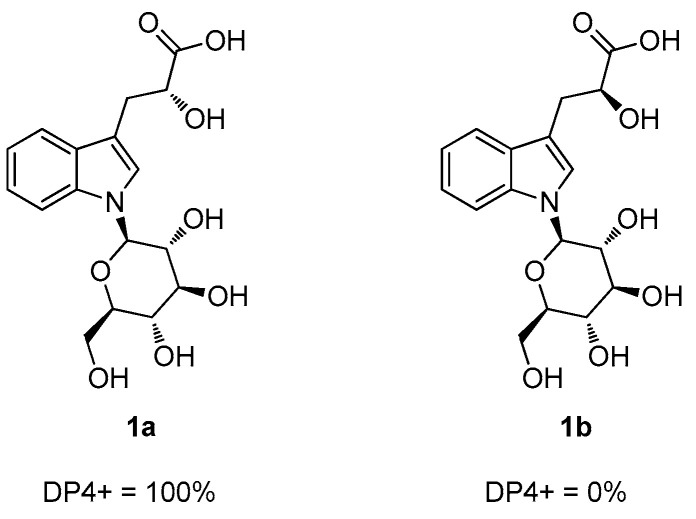
DP4+ analysis and probability scores for compound **1** with **1a**/**1b**.

Finally, the absolute configuration of C-11 in compound **1** was established by gauge-including atomic orbital NMR chemical shift calculations followed by DP4+ probability analysis [30,31]. The calculated ^1^H and ^13^C NMR chemical shifts for the two possible diastereomers, **1a** (11*R*) and **1b** (11*S*), were compared with the experimental values for compound **1** using DP4+ probability analysis. This analysis showed that compound **1a** (11*R*) had a DP4+ probability score of 100% (Figure 3). Based on these data, the chemical structure of compound **1** was determined to be D-indole-3-lactic acid *N*-*β*-D-glucopyranoside, as shown in Figure 1, and it was named deodeokaloid.

**Scheme 1:**
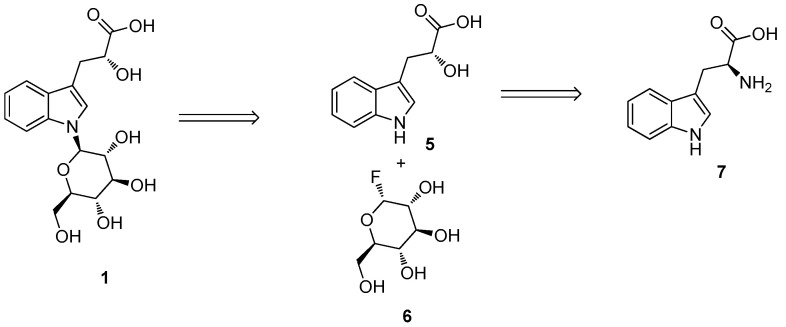
Retrosynthetic analysis of deodeokaloid.

Retrosynthetic analysis is crucial in the synthesis of natural products due to its ability to simplify complex molecular structures into manageable building blocks. This method aids chemists in planning efficient synthetic routes by deconstructing a target molecule into simpler components that lead to readily available starting materials. It identifies synthetic challenges, such as stereocenter formation and reaction selectivity, allowing for early modifications to avoid problematic steps. Moreover, retrosynthetic analysis drives innovation by revealing the need for new synthetic methods, optimizing cost and efficiency, and enabling the total synthesis of natural products when biological extraction is impractical. Additionally, it serves as an invaluable educational tool that enhances logical and creative thinking in chemical problem-solving. Overall, retrosynthetic analysis is indispensable for effectively understanding, manipulating, and synthesizing the complex structures of natural products. Thus, a retrosynthetic analysis of compound **1** was incorporated to provide valuable insights into potential synthetic strategies. As outlined in Scheme 1, our retrosynthetic analysis of deodeokaloid (**1**) involves a one-pot *N*-glycosylation of (*R*)-indole-3-lactic acid (**5**) and α-D-glucopyranosyl fluoride (**6**). (*R*)-Indole-3-lactic acid (**5**) can be derived from L-tryptophan (**7**) via the formation of alcohols from amines. 

The known compounds were identified as tangshenoside I (**2**) [32], tangshenoside IV (**3**) [33], and chlorogenic acid (**4**) [34] based on the comparison of their NMR spectral dataset with the reported data and LC/MS analysis.

### 2.2. Evaluation of Biological Activity of the Isolated Compounds

*Helicobacter pylori* is a widespread pathogen known to cause gastritis, duodenal ulcers, and gastric cancer [35]. Currently, treatment for *H. pylori* typically includes triple therapy, which involves a proton pump inhibitor combined with two antibiotics such as amoxicillin and clarithromycin or metronidazole. However, these treatments are often associated with side effects, including antibiotic resistance and gastrointestinal disturbances like nausea, vomiting, diarrhea, and abdominal pain [36]. As a result, there is increasing interest in natural products as adjuvant therapies with fewer adverse effects. Therefore, we evaluated the antimicrobial activity of isolated compounds **1**–**4** against *H. pylori* strain 51 (Table 2). An antibiotic in the clinical field, metronidazole, and an anti-*H. pylori* natural product, quercetin, [37], were used as positive controls. Among the compounds tested, compounds **2** and **3** demonstrated significant anti-*Helicobacter pylori* activity at a final concentration of 100 μM. The inhibitory activity of **3** (36.8 ± 0.6%) was more potent than that of **2** (22.2 ± 1.4%), which was comparable to that of quercetin (38.4 ± 2.3% inhibition), a positive control. Other compounds exhibited very weak inhibition. The syringin moiety in **3** may play a role in the difference in this anti-*H. pylori* activity between **2** and **3**, because syringin itself is known to have anti-*H. pylori* activity [38]. This is the first report on the anti-*H. pylori* activity of the four compounds.

Oxidative stress resulting from *H. pylori* infection contributes to chronic inflammation and gastric carcinogenesis [39]. The root of *C. lanceolata* has been reported to show antioxidant activity [40]. Thus, the isolated compounds **1**–**4** were evaluated for antioxidant activities. In an ABTS radical scavenging assay, compound **4** showed 1.6-fold higher antioxidant activity with the value of 1624.7 ± 135.2 mmol TE/mol than Trolox as a positive control. Compounds **1** and **2** displayed weak antioxidant activity with values of 82.7 ± 5.0 and 61.5 ± 2.6 mmol TE/mol, respectively. For the DPPH radical scavenging assay, all compounds exhibited the same pattern as the ABTS radical scavenging assay, but the values were lower than those in the ABTS radical scavenging assay. The antioxidant activity of chlorogenic acid (**4**) has been previously reported [41]. The Trolox equivalent value of chlorogenic acid in this study was similar to the previously reported result [42]. Oxidative stress generated by the excessive production of reactive oxygen species (ROS) is associated with inflammatory response signaling pathways, damaging cell membranes, lipids, nucleic acids, and proteins [43]. This leads to various diseases including cancer, dementia, and diabetes. Therefore, we evaluated the cellular antioxidant capacity of the isolated compounds using DCFH_2_-DA in LPS-stimulated RAW 264.7 cells. Compounds **2**, **3**, and **4** significantly reduced LPS-mediated ROS production at a concentration of 100 μM, with the values of 34.9 ± 5.6%, 25.5 ± 5.3%, and 21.9 ± 6.2%, respectively. Compound **1** failed to show the cellular antioxidant activity.

## 3. Materials and Methods

### 3.1. Equipment Used for Analyses

The equipment and devices used in the analyses and experiments are listed in Table S1.

### 3.2. Plant Material

**Figure 4:**
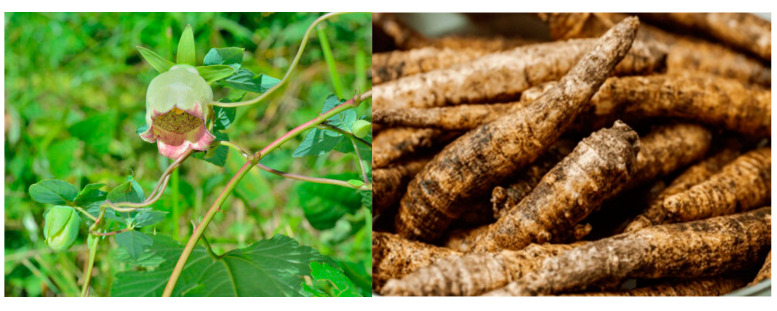
Photographs of aerial parts and roots of *C. lanceolata*.

The dried roots of *C. lanceolata* were sourced from Donghoengseong Agricultural Cooperation in Gangwon-do, Korea in October 2018. The plant material was authenticated by one of the authors (K. H. Kim). A voucher specimen has been deposited in the herbarium of the School of Pharmacy, Sungkyunkwan University, Suwon, Korea (Figure 4).

### 3.3. Extraction and Isolation

Dried *C. lanceolata* roots (3.5 kg) were extracted using 50% ethanol (20 L) at 50 °C for 20 h in an herb extractor. The extract was then concentrated under reduced pressure using a rotary evaporator and further dried at 50 °C for three days, yielding 104.3 g of crude ethanol extract. This extract was suspended in 700 mL of distilled water and subjected to sequential solvent partitioning with *n*-hexane, dichloromethane (CH_2_Cl_2_), ethyl acetate (EtOAc), and *n*-butanol (*n*-BuOH), each solvent used three times with 700 mL, resulting in four fractions weighing 9.2 g, 3.1 g, 2.7 g, and 20 g, respectively.

Analysis of LC/MS and HR/MS data from the fractions indicated that the *n*-BuOH fraction contained significant compounds, including alkaloids, phenolic glycosides such as tangshenosides, and phenylpropanoids. The *n*-BuOH fraction (20 g) was dissolved in distilled water, loaded onto a Diaion HP-20 column, and eluted with distilled water followed by methanol, producing two fractions. The methanol-soluble fraction (9.1 g) was further separated using silica open column chromatography with a gradient solvent system [EtOAc-MeOH-H_2_O (9:3:1) → CH_2_Cl_2_-MeOH (1:1)], yielding eight fractions (DD0–DD7). Fraction DD4 (2.98 g) underwent Sephadex LH-20 column chromatography with a CH_2_Cl_2_-MeOH (2:8) solvent system, producing twelve sub-fractions (DD41–DD412). Sub-fraction DD48 (512 mg) was further purified using preparative HPLC with a 40% MeOH isocratic solvent system to produce four additional sub-fractions (DD481–DD484). Sub-fraction DD482 (105 mg) was isolated by semi-preparative HPLC with a C18 column and a 27% MeOH isocratic solvent system, yielding compound **1** (*t*_R_ = 26 min, 10.8 mg) and compound **4** (*t*_R_ = 24 min, 6.0 mg). Finally, fraction DD5 (534.4 mg) was processed through preparative HPLC using a gradient from 40% to 100% MeOH, resulting in six sub-fractions (DD51–DD56). Sub-fraction DD52 (43.9 mg) was isolated using semi-preparative HPLC with a C18 column and a gradient solvent system from 25% to 50% MeOH, yielding compound **2** (*t*_R_ = 41 min, 13 mg) and compound **3** (*t*_R_ = 71 min, 0.3 mg).

#### Deodeokaloid (**1**)

Colorless gum; [${\alpha ]}_{D}^{25}$-21.4 (*c* 0.05, MeOH); UV (MeOH): λ_max_ (log *ε*) = 225 (4.2) nm; ECD (MeOH) *λ*_max_ (Δ*ε*) 229 (−1.63), 258 (−0.18), 283 (0.09); IR (neat): *ν*_max_ = 3351, 2931, 2854, 1698, 1445, 1372, 1220, 1081, 1023 cm^−1^; ^1^H (850 MHz) and ^13^C (212.5 MHz) NMR data, see Table 1; HR-ESIMS (positive-ion mode) *m/z* 368.1351 [M + H]^+^ (calcd. for C_17_H_22_NO_8_, 368.1345).

### 3.4. Enzymatic Hydrolysis and Sugar Identification of ***1***

To determine the absolute configuration of the sugar moiety in compound **1** [44], 4.0 mg of the compound was hydrolyzed using β-glucosidase (8.0 mg) from almonds, which was dissolved in distilled water. The hydrolysis reaction was performed at 37 °C for 72 h in a dry oven. Upon cooling, the aglycone was extracted through solvent partitioning using EtOAc. The aqueous layer was then evaporated under vacuum, redissolved in 0.4 mL of anhydrous pyridine, and treated with 1 mg of L-cysteine methyl ester chloride. The mixture was heated at 60 °C for 1 h, followed by the addition of 30 μL of *O*-tolylisothiocyanate, and heated for another hour at the same temperature. The reaction mixture was concentrated, redissolved in methanol, and analyzed by LC/MS on a C18 column using a gradient solvent system of 35% to 65% methanol. For comparison, standard substances of D-glucose and L-glucose underwent the same derivatization and were analyzed under identical conditions. Based on the retention times in the LC/MS analysis, the sugar moiety in compound **1** was identified as D-glucose, with compound **1** showing a retention time of 5.9 min, compared to 6.2 min for D-glucose and 1.7 min for L-glucose.

### 3.5. Computational NMR Chemical Shift Calculations for DP4+ Analysis

The DP4+ probability analysis was carried out on the geometrically optimized conformers of diastereomers **1a** and **1b**. Calculations were conducted at the B3-LYP/6-31+G(d,p) level [45], utilizing an Excel sheet (DP4+) provided by Grimblat et al. [46] for calculating the GIAO magnetic shielding tensors.

Chemical shift values were derived from these magnetic shielding tensors by using the following equation, where *δ*$\begin{array}{c} x\\ calc \end{array}$ refers the calculated NMR chemical shift for nucleus *x*, and *σ*^o^ represents to the shielding tensor for the proton and carbon nuclei in tetramethylsilane:

$$\delta_{calc}^{x}=\sigma^{o}-\sigma^{x}$$

The density-functional theory using the B3-LYP/6-31+G(d,p) basis set was applied for the shielding tensor calculations.

The unscaled NMR properties of the optimized structures were averaged, and the scaled chemical shift values were then calculated using the following:

$$\delta_{scaled}=\frac{\delta_{unscaled}-intercept}{slope}$$

This approach ensures precision in aligning the theoretical chemical shifts with experimental data, providing a robust method for confirming molecular structures.

### 3.6. Anti-Helicobacter Pylori Activity

The *H. pylori* Korean Type Culture Collection at the School of Medicine, Gyeongsang National University, Korea provided a clinical strain of *H. pylori* 51, which was isolated from a Korean patient with a duodenal ulcer (HPKTCC B0006). The strain was cultured and maintained on Brucella broth medium (BD Co., Sparks, MD, USA) supplemented with 10% horse serum (Gibco, New York, NY, USA). Culture conditions were maintained at 37 °C, 100% humidity, and 10% CO_2_. The antibacterial activity was tested using a previously described method [47]. Briefly, 20 μL of bacterial colony suspension, equivalent to 2–3 × 10^8^ cfu/mL, was added to each well of a six-well plate containing Brucella broth medium supplemented with 10% horse serum. The final concentration of the test samples and the positive control was set at 100 μM, with a total volume of 2 mL per well. After 24 h of incubation, bacterial growth was assessed by measuring the optical density at 600 nm using an Optizen POP UV/VIS spectrophotometer (Mecasys, Daejeon, Republic of Korea). Anti-*H. pylori* activity was calculated using the following equation:

Inhibition (%) = [(absorbance of the control − absorbance of solution with samples)/absorbance of the control] × 100.

DMSO served as the negative control, while quercetin and metronidazole (Sigma, St. Louis, MO, USA) were used as positive controls.

### 3.7. Antioxidant Activity

The ABTS radical was generated using a method previously described [48,49]. For the assay, 20 μL of each sample was mixed with 180 μL of the ABTS•+ solution and incubated at room temperature. After 10 min, the absorbance was measured at 734 nm. The antioxidant activity of each sample was quantified and expressed as Trolox equivalents (Sigma, St. Louis, MO, USA) per gram (μmol TE/g). Another antioxidant activity was evaluated with DPPH radicals generated by our previously reported methods [50,51]. The same volume of 0.2 mM DPPH•+ radical solution was added to each sample and incubated at room temperature for 10 min. The absorbance was measured at 520 nm, and the DPPH radical scavenging activity was calculated as Trolox equivalents.

### 3.8. Determination of Intracellular ROS Scavenging Activity

Intracellular ROS levels were measured by the dichlorodihydrofluorescein diacetate (DCFH_2_-DA) assay using our previously reported method [52]. RAW 264.7 cells (1 × 10^5^ cells/well) were seeded into a 96-well plate and incubated overnight to allow for adhesion. After this period, the cells were treated with compounds **1–4** and lipopolysaccharide (LPS). Following a 24 h incubation, the supernatants were removed and the cells were reacted with DCFH_2_-DA reagents (Sigma, St. Louis, MO, USA). After 30 min in the 5% CO_2_ incubator at 37 °C, the fluorescence was measured by a Victor X5 multilabel plate reader (Perkin Elmer, Waltham, MA, USA) (Excitation, 485 nm; Emission, 535 nm).

### 3.9. Statistical Analysis

All contents are expressed as means ± standard deviations (SD) based on triplicate determinations. Differences among samples were statistically evaluated using one-way analysis of variance (ANOVA), with significance assessed at the 5% level using two-sided tests.

## 4. Conclusions

In this study, we investigated potential bioactive compounds from *C. lanceolata* root extracts, which led to the isolation and identification of a new indole alkaloid *N*-glycoside, deodeokaloid (D-indole-3-lactic acid *N*-β-D-glucopyranoside) (**1**), alongside known compounds tangshenoside I (**2**), tangshenoside IV (**3**), and chlorogenic acid (**4**). The structure of the new compound was determined using 1D and 2D NMR techniques, HR-ESIMS, DP4+ probability analysis, and chemical reactions. We evaluated the isolated compounds for their anti-*H*. *pylori* and antioxidant activities. Notably, compound **3** showed significant anti-*H. pylori* activity similar to that of quercetin as the positive control. Compound **4** exhibited potent antioxidant activities in ABTS and DPPH radical scavenging assays. Compounds **2**–**4** displayed significant intracellular ROS scavenging capacity. These biological activities of the isolates help to elucidate the various physiological activity mechanisms of *C. lanceolate* roots. In addition, this study highlights *C. lanceolata* roots as a promising natural source for bioactive compounds with potential therapeutic applications.

## Figures and Tables

**Table 1:** ^1^H (850 MHz) and ^13^C NMR (212.5 MHz) data for compound **1** in CD_3_OD (*δ* ppm) *^a^.*

Position	1	
	*δ*_H_ (*J* in Hz)	*δ*_C_
2	7.31, s	125.4, CH
3		112.7, C
4	7.59, d (8.0)	119.9, CH
5	7.06, t (8.0)	120.7, CH
6	7.15, t (8.0)	122.8, CH
7	7.50, d (8.0)	111.4, CH
8		138.3, C
9		130.2, C
10	3.24, d (14.0); 3.09, dd (14.0, 7.0)	31.4, CH_2_
11	4.41, br s	71.9, CH
12		179.1, C
1′	5.42, d (9.0)	86.7, CH
2′	3.92, t (9.0)	73.7, CH
3′	3.60, t (9.0)	79.0, CH
4′	3.49, t (9.0)	71.5, CH
5′	3.56, ddd (9.0, 6.0, 2.0)	80.5, CH
6′	3.70, dd (12.0, 6.0); 3.87, dd (12.0, 2.0)	62.8, CH_2_

*^a^* Coupling constants (Hz) are given in parentheses, and ^13^C NMR data were assigned based on HSQC and HMBC experiments.

**Table 2:** Anti-*H. pylori* activity of compounds **1**–**4** against *H. pylori* strain 51 treated with 100 μM of each compound.

Compound	Inhibition (%) **
1	13.4 ± 0.9 ^d^
2	22.2 ± 1.4 ^c^
3	36.8 ± 0.6 ^b^
4	11.9 ± 0.9 ^d^
Quercetin *	38.4 ± 2.3 ^b^
Metronidazole *	96.6 ± 0.5 ^a^

* Positive controls. ** Different letters in the same column mean significantly different by Duncan’s multiple range test (*p* < 0.05).

## Data Availability

Data is contained within the article and Supplementary Materials.

## Supplementary Materials

The following are available online at https://www.mdpi.com/article/10.3390/plants13223243/s1, Figure S1: The HR-ESIMS data of compound **1**; Figure S2: The ESI-MS data of compound **1**; Figure S3: The UV spectrum of **1**; Figure S4: The ^1^H NMR spectrum of compound **1** (CD_3_OD, 850 MHz); Figure S5: The ^13^C NMR spectrum of compound **1** (CD_3_OD, 212.5 MHz); Figure S6: The ^1^H-^1^H COSY spectrum of compound **1**; Figure S7: The NOESY spectrum of compound **1**; Figure S8: The HSQC spectrum of compound **1**; Figure S9: The HMBC spectrum of compound **1**; Figure S10: DP4+ analysis of compound 1 with isomers **1a** and **1b**; Table S1: Equipment used for analyses; Table S2: Gibbs free energies and Boltzmann distribution of conformers of **1a**; Table S3: Gibbs free energies and Boltzmann distribution of conformers of **1b**; Table S4: Coordinates of the conformers of **1a** and **1b**
