# Supplementary material for: Deodeokaloid, a New Indole Alkaloid N-Glycoside and Bioactive Phenolic Compounds from the Roots of Codonopsis lanceolata
Source: Plants (Basel). 2024 Nov 19;13(22):3243. doi: 10.3390/plants13223243 (PMC11598484; doi:10.3390/plants13223243)
Supplement: Supplementary file 1 [file plants-13-03243-s001.zip › plants-3300574-supplementary.pdf]

Supplementary Materials

Deodeokaloid, a New Indole Alkaloid *N*-Glycoside and Bioactive Phenolic Compounds from the Roots of *Codonopsis lanceolata*

Yeo Rang Cho [1], Joo-Hyun Hong [1], Dong-Min Kang [2], Yoon-Joo Ko [3], Mi-Jeong Ahn [2,*], Ki Hyun Kim [1,*]

[1]School of Pharmacy, Sungkyunkwan University, Suwon 16419, Republic of Korea; yr6755@g.skku.edu (Y.R.C.); ehong@skku.edu (J.H.H.)

[2]College of Pharmacy and Research Institute of Pharmaceutical Sciences, Gyeongsang National University, Jinju 52828, Republic of Korea; kdm7105@gnu.ac.kr (D.-M.K.)

[3]Laboratory of Nuclear Magnetic Resonance, National Center for Inter-University Research Facilities (NCIRF), Seoul National University, Gwanak-gu, Seoul 08826, Republic of Korea; yjko@snu.ac.kr (Y.J.K.)

*Correspondence: khkim83@skku.edu (K.H.K.); +82-31-290-7700 (K.H.K.); amj5812@gnu.ac.kr (M.-J.A.)

[1]These authors contributed equally.

Supplementary Materials Contents

Figure S1. The HR-ESIMS (positive ion mode) data of **1** S3

Figure S2. ESI-MS (positive ion mode) data of **1** S4

Figure S3. The UV spectrum of **1** S5

Figure S4. The ^{1}H NMR spectrum of **1** (CD_3OD, 850 MHz) S6

Figure S5. The ^{13}C NMR spectrum of **1** (CD_3OD, 212.5 MHz) S7

Figure S6. The ^{1}H-^{1}H COSY spectrum of **1** S8

Figure S7. The NOESY spectrum of **1** S9

Figure S8. The HSQC spectrum of **1** S10

Figure S9. The HMBC spectrum of **1** S11

Figure S10. DP4+ analysis of compound **1** with isomers **1a** and **1b** S12

Table S1. Equipment used for analyses S13

Table S2. Gibbs free energies and Boltzmann distribution of conformers **1a** S15

Table S3. Gibbs free energies and Boltzmann distribution of conformers **1b** S16

Table S4. Coordinates of the conformers of **1a** and **1b** S17

Figure S1. The HR-ESIMS (positive ion mode) data of **1**

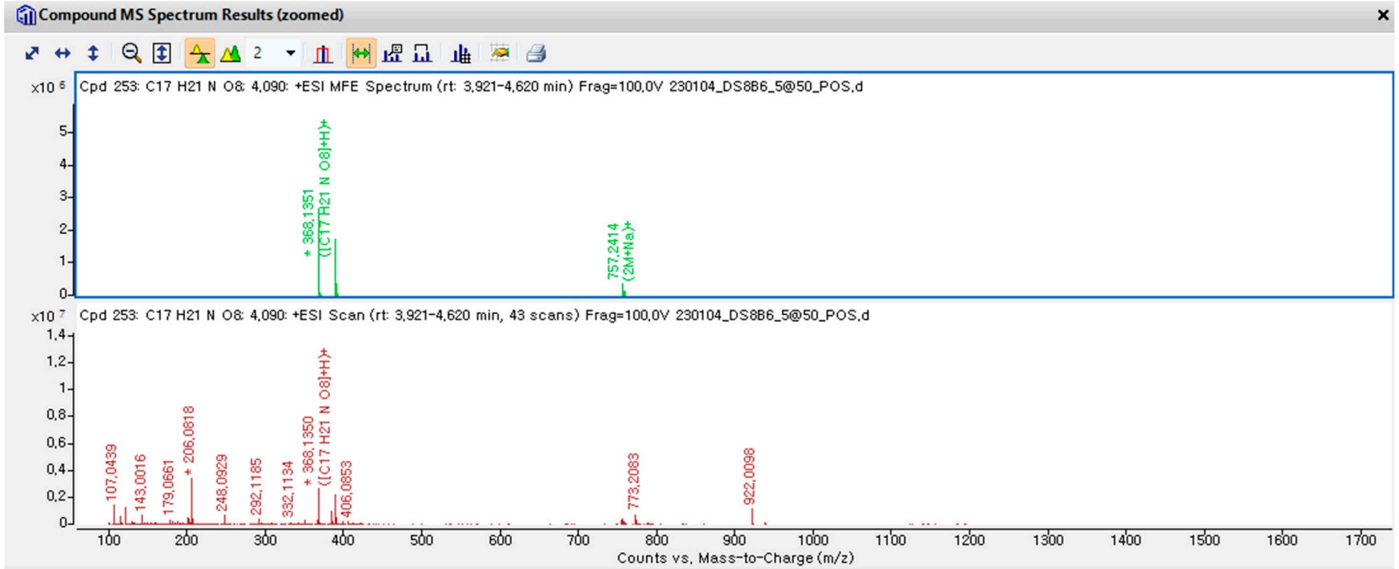

Compound Identification Results: Cpd 253: C17 H21 N O8; 4.090

ID Techniques Applied
MFG

Best	Name	Formula	m/z	Mass	Mass (Tgt)	Diff (ppm)	Score (Tgt)	RT	RT (Tgt)	RT Diff	Score (RT)	Species	Score (DB)	Score (Lib)	Score	Flags
○		C18 H17 N5 O4	368.1351 390.1169	367.1277		-0.86		4.09				(M+H)+ (M+Na)+			99.19	
⊙		C17 H21 N O8	368.1351 390.1169	367.1277		2.57		4.09				(M+H)+ (M+Na)+			96.98	
○		C16 H15 N8 O3	368.1351 390.1169	367.1278		3.02		4.09				(M+H)+ (M+Na)+			95.76	
○		C20 H19 N2 O5	368.1351 390.1169	367.1278		-4.38		4.09				(M+H)+ (M+Na)+			91.82	
○		C19 H13 N9	368.1351 390.1169	367.1278		-4.31		4.09				(M+H)+ (M+Na)+			90.44	

Figure S2. ESI-MS (positive ion mode) data of **1**

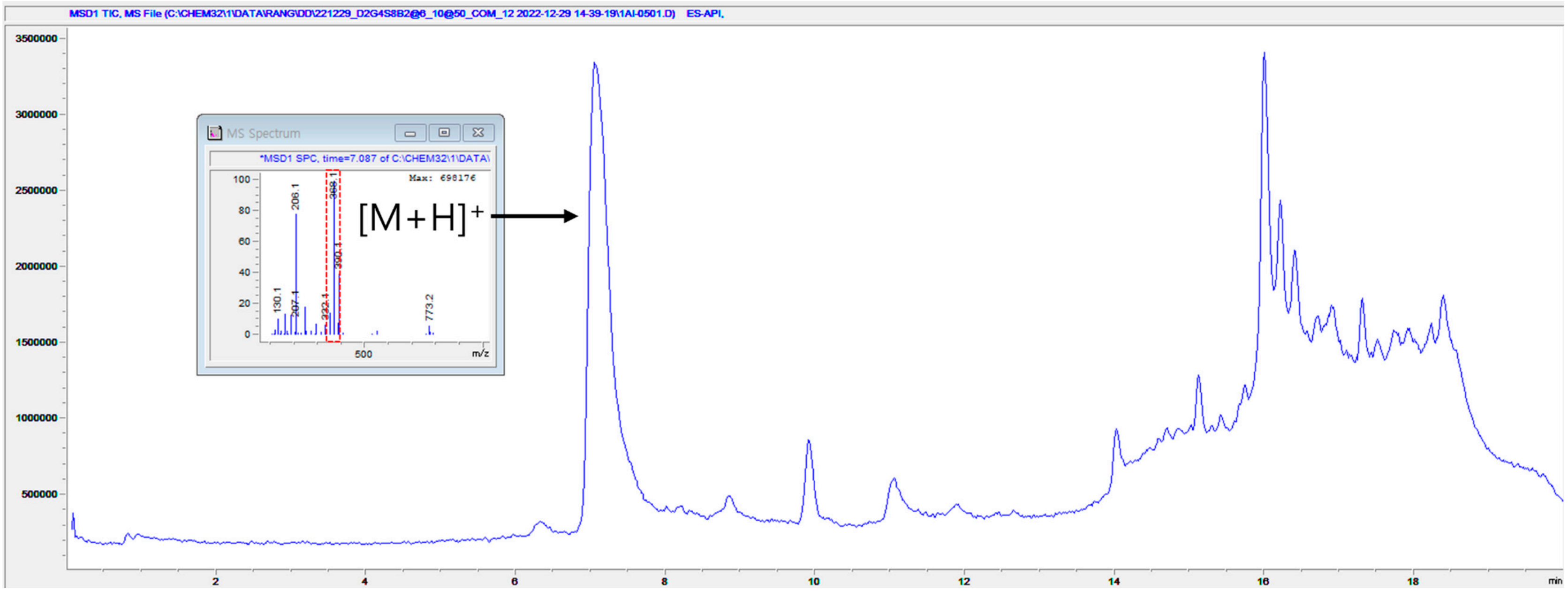

Figure S3. The UV spectrum of **1**

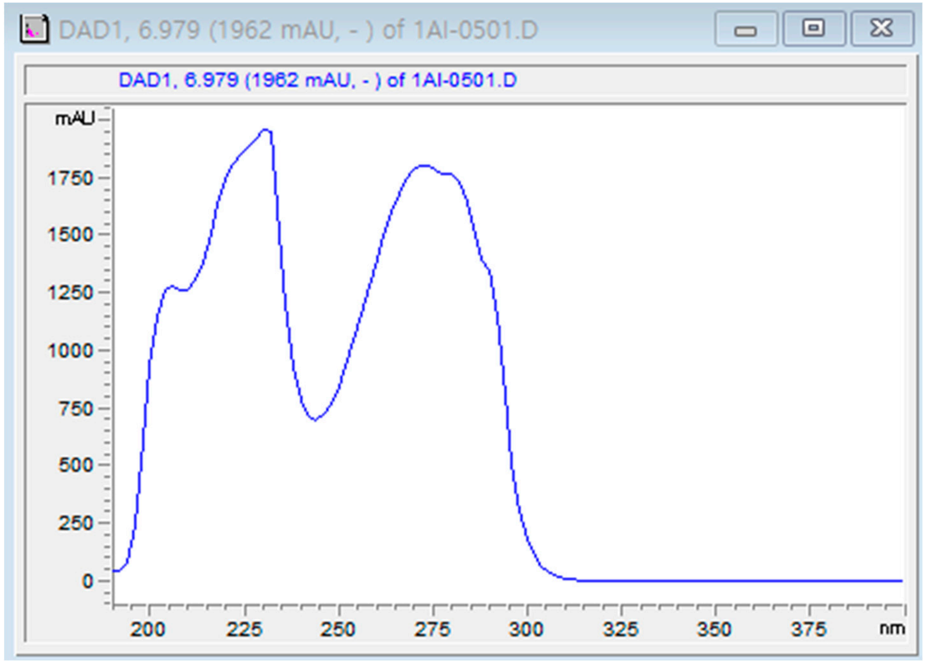

Figure S4. The ^{1}H NMR spectrum of **1** (CD_3OD, 850 MHz)

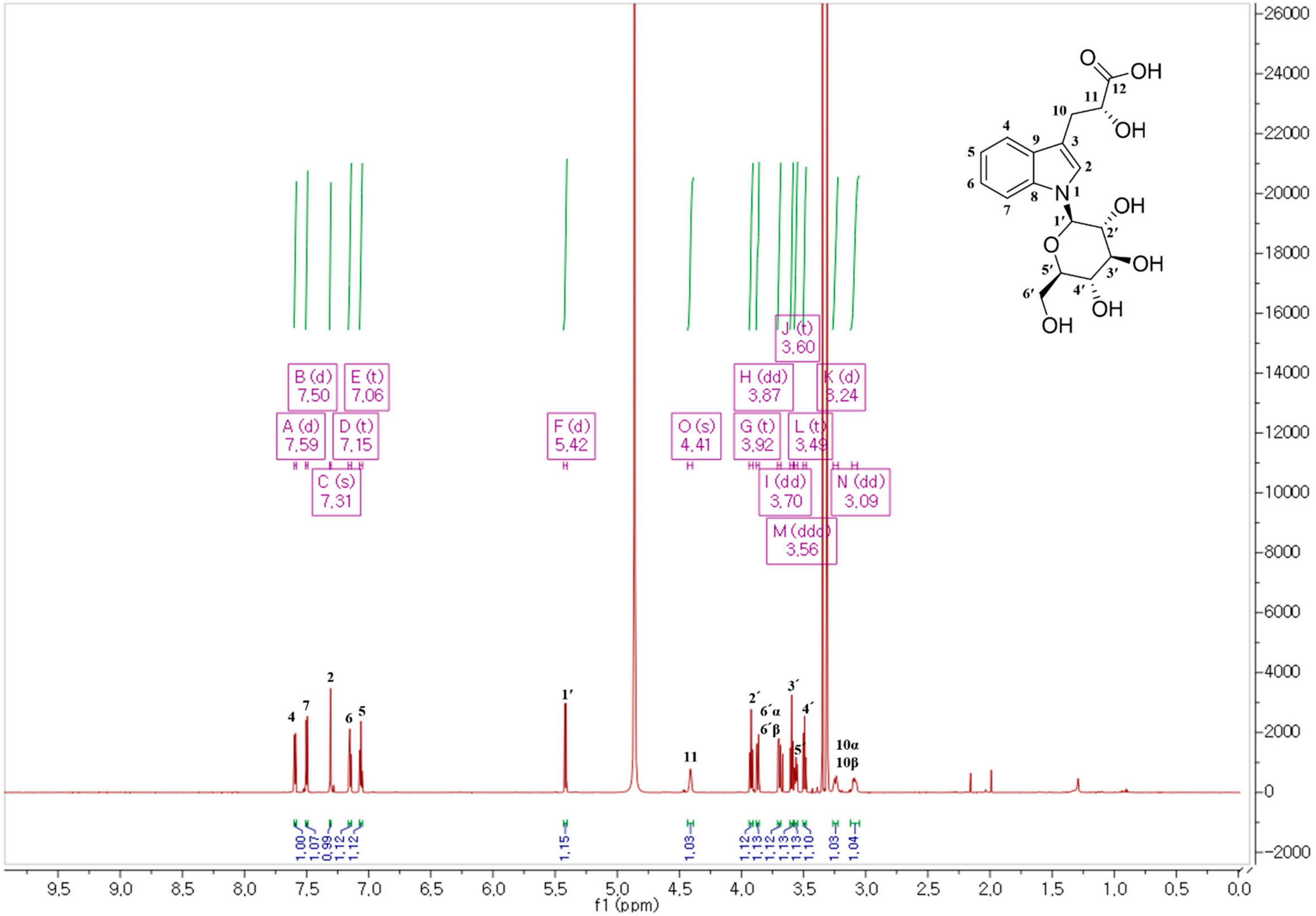

Figure S5. The ^{13}C NMR spectrum of **1** (CD_3OD, 212.5 MHz)

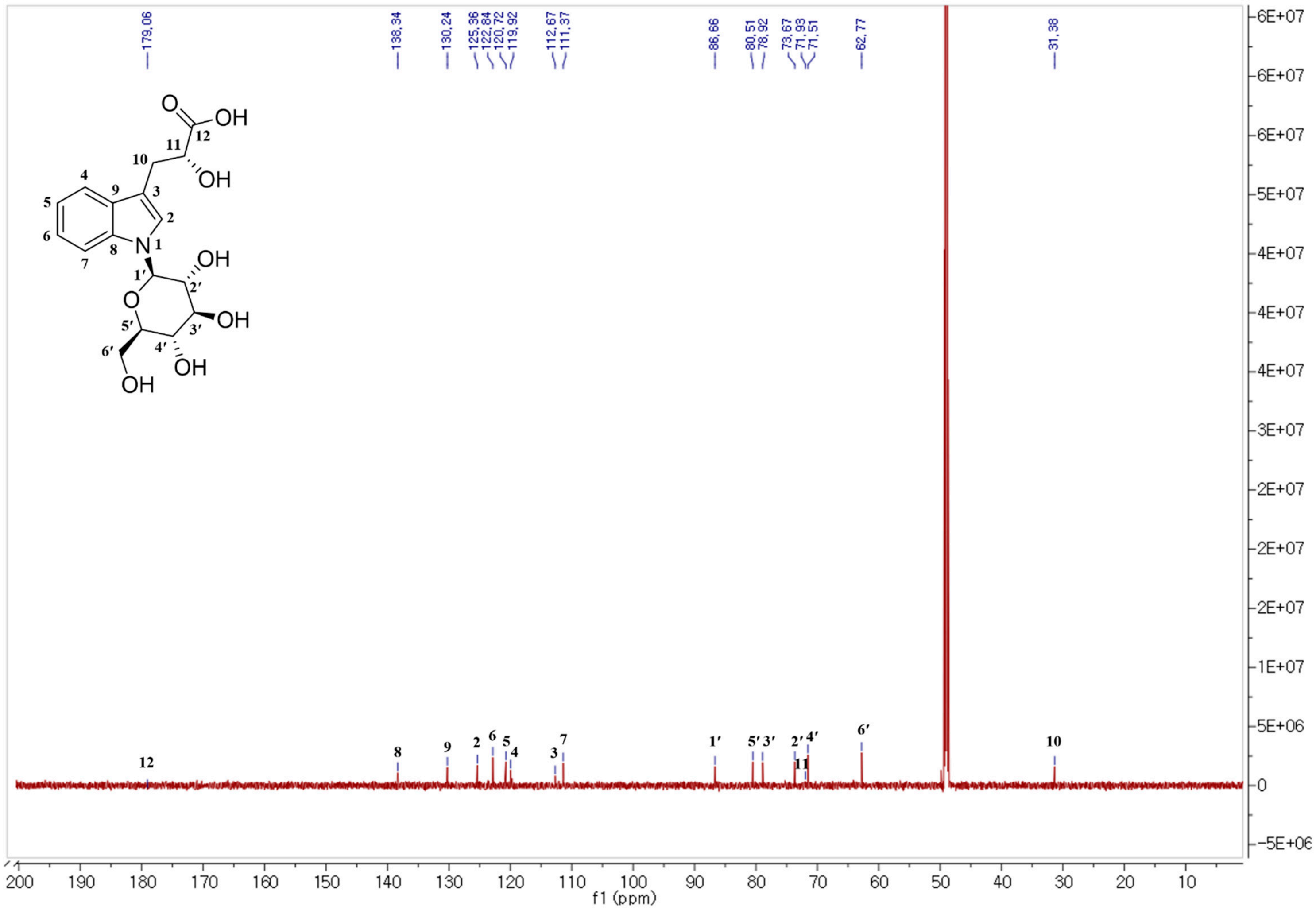

Figure S6. The ^{1}H-^{1}H COSY spectrum of **1**

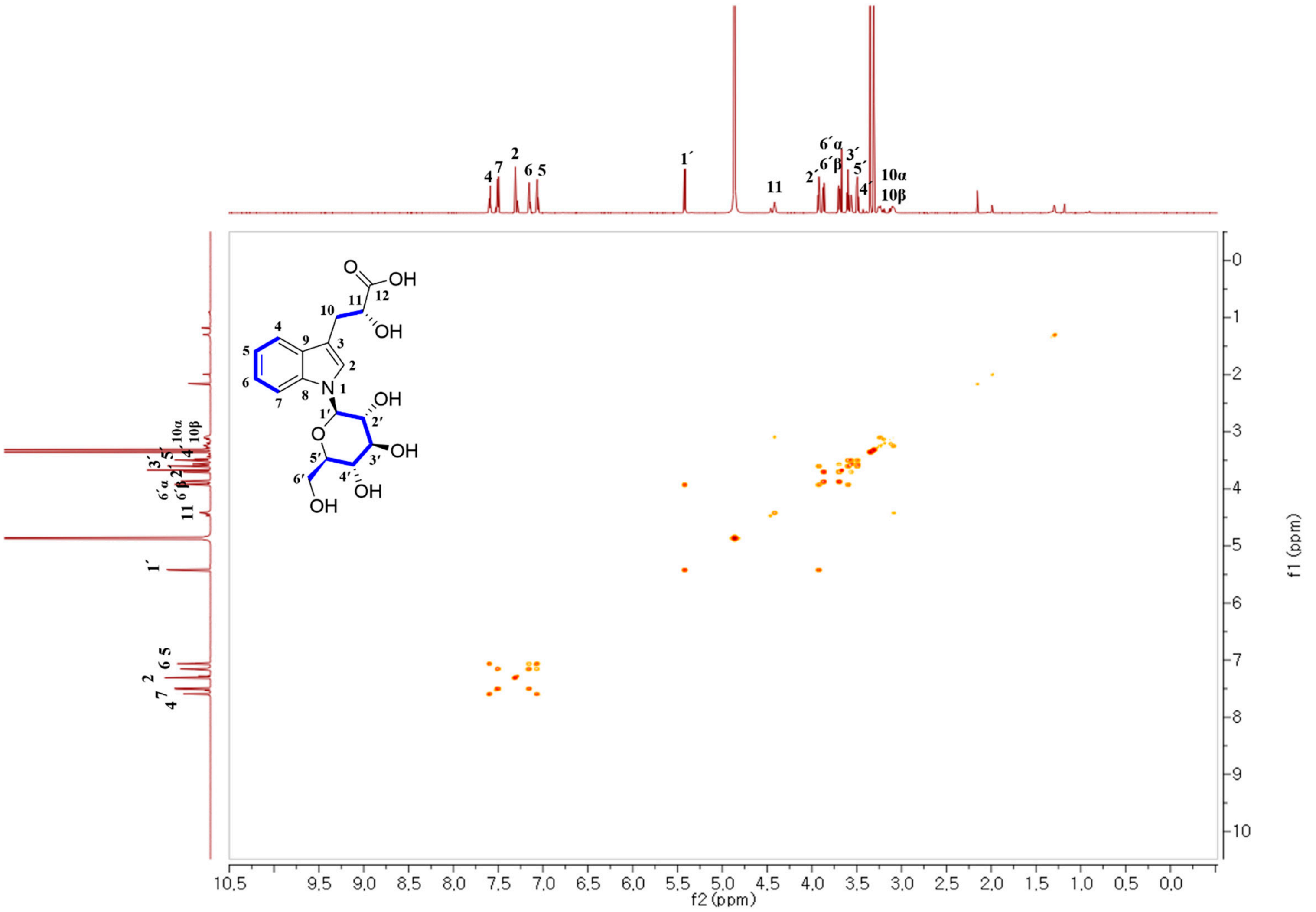

Figure S7. The NOESY spectrum of **1**

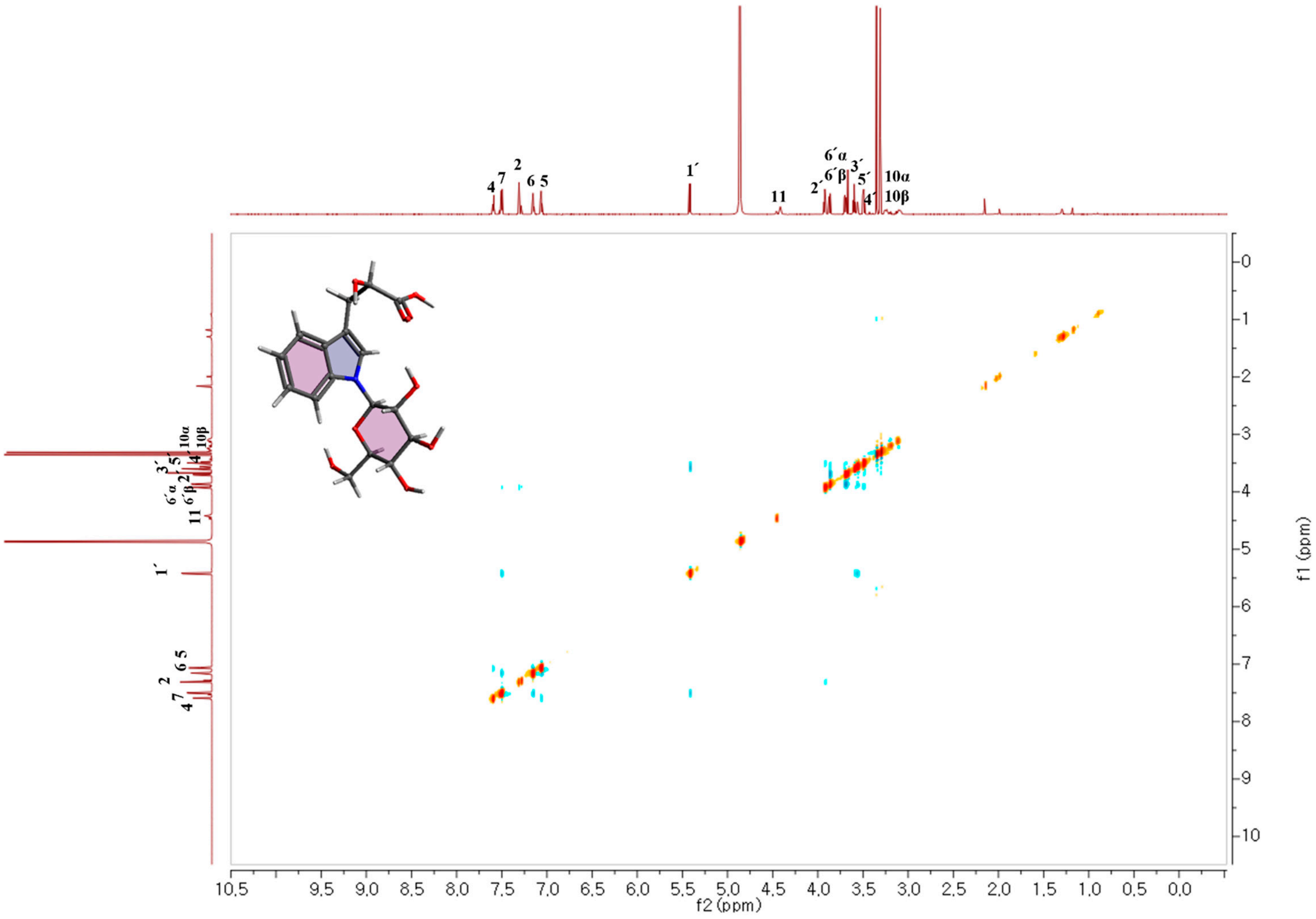

Figure S8. The HSQC spectrum of **1**

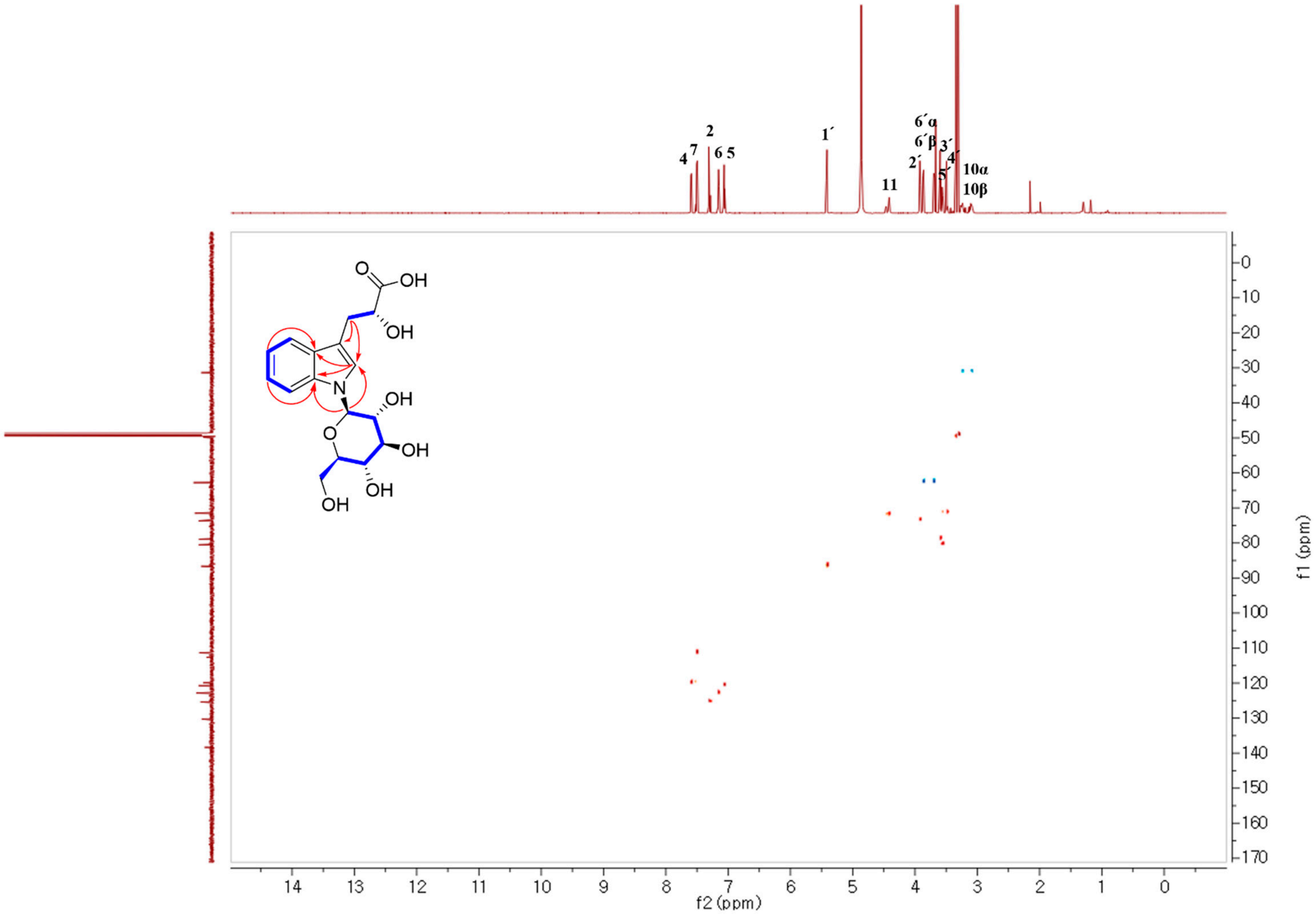

Figure S9. The HMBC spectrum of **1**

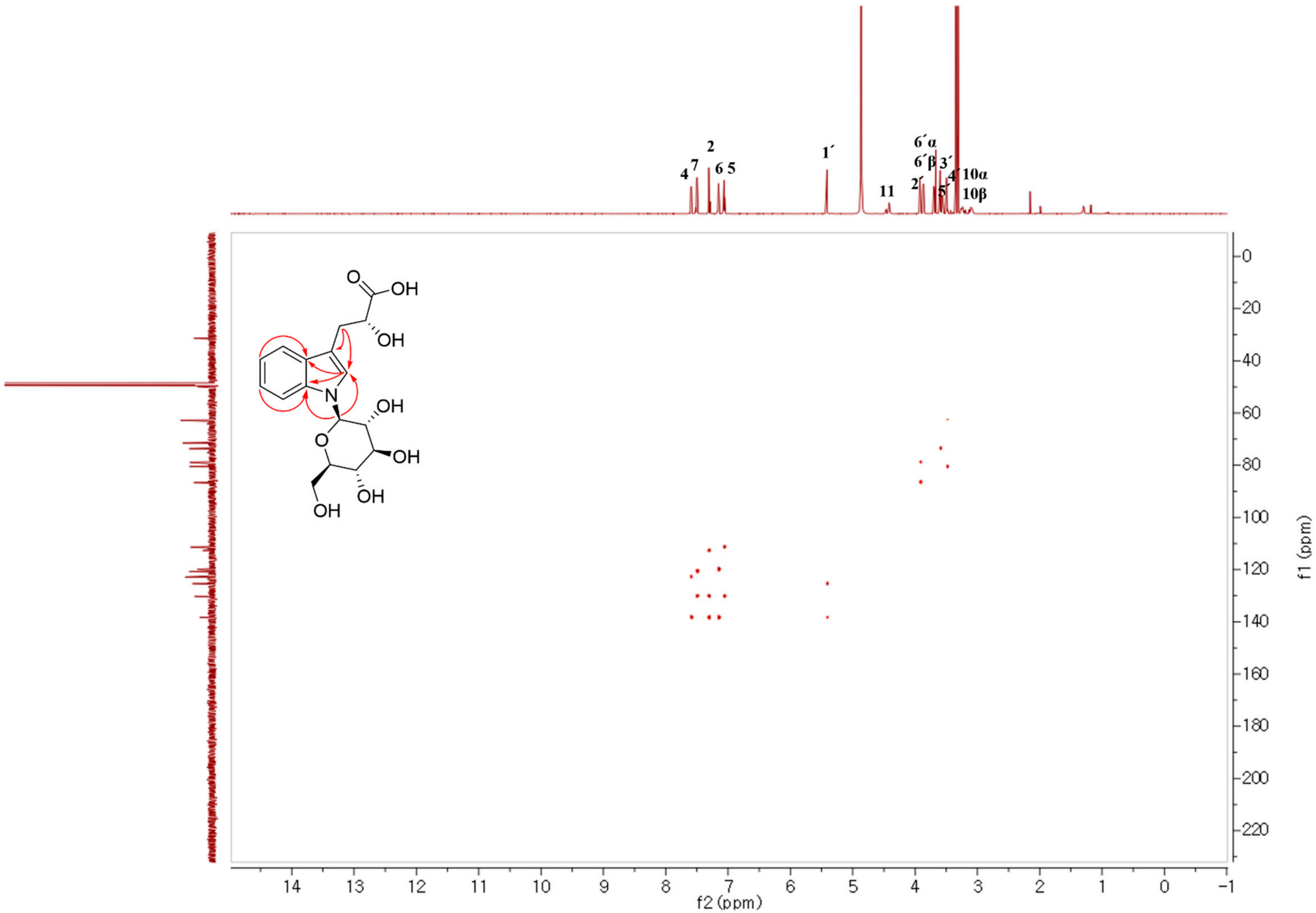

Figure S10. DP4+ analysis and probability scores for **1** with **1a/1b**

Functional	Solvent?	
B3LYP	PCM	
	Isomer 1	Isomer 2
sDP4+ (H data)	0.00%	100.00%
sDP4+ (C data)	95.87%	4.13%
sDP4+ (all data)	0.00%	100.00%
uDP4+ (H data)	99.14%	0.86%
uDP4+ (C data)	100.00%	0.00%
uDP4+ (all data)	100.00%	0.00%
DP4+ (H data)	0.02%	99.98%
DP4+ (C data)	100.00%	0.00%
DP4+ (all data)	100.00%	0.00%
	**1a**	**1b**

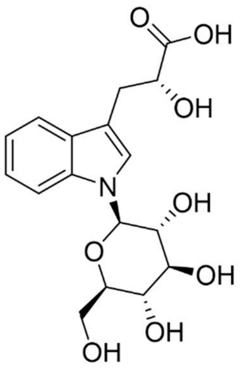

1a

DP4+ = 100%

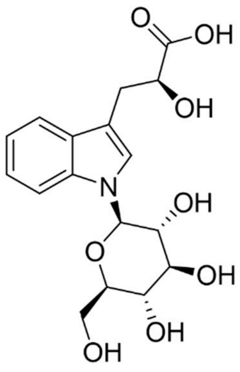

1b

DP4+ = 0%

Table S1. Equipment used for analyses

Experimental procedure	Equipment
Optical rotations	JASCO P-2000 polarimeter (JASCO, Easton, MD, USA)
Ultraviolet (UV) spectra	Agilent 8453 UV-visible spectrophotometer (Agilent Technologies, Santa Clara, CA, USA)
Electronic circular dichroism (ECD) spectra	JASCO J-1500 spectropolarimeter (JASCO, Easton, MD, USA)
Infrared (IR) spectra	Bruker IFS-66/S FT-IR spectrometer (Bruker, Karlsruhe, Germany)
Nuclear magnetic resonance (NMR) spectra	Bruker AVANCE III HD 850 NMR spectrometer with a 5 mm TCI CryoProbe operating at 850 MHz (^{1}H) and 212.5 MHz (^{13}C)
HR-ESIMS	•Agilent G6545B quadrupole time-of-flight mass spectrometer (Agilent Technologies)<br>•Agilent 1290 Infinity II high-performance liquid chromatography (HPLC) instrument (Agilent Eclipse Plus C18 column (2.1 × 50 mm, 1.8 µm; flow rate: 0.3 mL/min))
Preparative HPLC	Waters 1525 Binary HPLC pump with a Waters 996 Photodiode Array Detector (Waters Corporation, Milford, MA, USA) and a Hector C18 column (250 × 21.2 mm, 5 µm; flow rate: 5 mL/min; Rstech Corporation, Korea)

Semi-preparative HPLC	Waters 1525 Binary HPLC pump with a Waters 996 Photodiode Array Detector (Waters Corporation, Milford, CT, USA)<br>•Phenomenex Luna C18 column (250 × 10 mm, 10 µm; flow rate: 2 mL/min; Phenomenex, Torrance, CA, USA)<br>•Phenomenex Luna Phenyl-Hexyl column (250 × 10 mm, 10 µm; flow rate: 2 mL/min; Phenomenex)
LC/MS analysis	Agilent 1200 Series HPLC system equipped with a diode array detector and 6130 Series ESI mass spectrometer using an analytical Kinetex C18 100 Å column (100 × 2.1 mm, 5 µm; flow rate: 0.3 mL/min; Phenomenex).
Column chromatography	•Silica gel 60 (230-40 mesh; Merck, Darmstadt, Germany)<br>•RP-C18 silica gel (Merck, 230-240 mesh)<br>•Sephadex LH-20 (Pharmacia, Uppsala, Sweden)<br>•Diaion HP20 (Mitsubishi Chemical, Tokyo, Japan)
Thin-layer chromatography (TLC)	pre-coated silica gel F254 plates and RP-C18 F254s plates (Merck); spots were detected under UV light or by heating following spraying with anisaldehyde-sulfuric acid.

Table S2. Gibbs free energies and Boltzmann distribution of conformers **1a**

Conformers	B3LYP/6-31+G(d,p) Gibbs free energy (298.15 K)		
	G (Hartree)	ΔG (kcal/mol)	Boltzmann distribution (%)
**1a**-1	-1316.268406	0.00	0.56%
**1a**-2	-1316.273252	-3.04	95.42%
**1a**-3	-1316.265645	1.73	0.03%
**1a**-4	-1316.270249	-1.16	3.97%
**1a**-5	-1316.265148	2.04	0.02%

Table S3. Gibbs free energies and Boltzmann distribution of conformers **1b**

Conformers	B3LYP/6-31+G(d,p) Gibbs free energy (298.15 K)		
	G (Hartree)	ΔG (kcal/mol)	Boltzmann distribution (%)
**1b**-1	-1316.268670	0.00	61.21%
**1b**-2	-1316.265501	1.99	2.13%
**1b**-3	-1316.268144	0.33	35.06%
**1b**-4	-1316.265225	2.16	1.59

Table S4. Coordinates of the conformers **1a** and **1b**

1a-1

Coordinates (Angstroms)			atom	charge
X	Y	Z		
1.22547007	6.49329927	0.64008978	c	6.00
-0.28703364	4.33404426	0.38309737	c	6.00
-2.9655468	4.47801641	0.20595116	c	6.00
-4.13368733	6.86507916	0.24998165	c	6.00
-2.63805405	9.00961024	0.48902872	c	6.00
0.01206605	8.82418217	0.68503541	c	6.00
-3.90473398	1.91845036	0.03358287	c	6.00
-1.82722198	0.36948702	0.10123572	c	6.00
0.37325342	1.79065099	0.28687934	n	7.00
-6.60103553	1.0527935	-0.17115506	c	6.00
-7.84312815	1.47517971	-2.79257064	c	6.00
-6.28276222	0.25686795	-4.88132792	c	6.00
-6.09987145	-2.27095888	-4.64996987	o	8.00
-5.28945947	1.45030676	-6.56862214	o	8.00

2.88923041	0.75657991	0.50393603	c	6.00
-8.182276	4.05583975	-3.34022261	o	8.00
3.05369278	-1.32772995	-1.17282964	o	8.00
5.51860936	-2.47993282	-1.26819206	c	6.00
6.21732682	-3.5130183	1.34404082	c	6.00
6.08179895	-1.37905403	3.28142106	c	6.00
3.49506246	-0.11167606	3.22190252	c	6.00
3.61485579	1.96106467	4.92238722	o	8.00
8.68408788	-4.53991631	1.21176018	o	8.00
6.6309584	-2.43824833	5.68415927	o	8.00
5.34916058	-4.49281598	-3.31995317	c	6.00
4.71713906	-3.42255746	-5.6860946	o	8.00
3.26406436	6.36807104	0.82723803	h	1.00
-6.16947631	7.00899439	0.0526305	h	1.00
-3.51962319	10.86337706	0.52399483	h	1.00
1.1333838	10.53291026	0.87769338	h	1.00
-1.71492724	-1.66540518	-0.01509848	h	1.00
-6.72321421	-0.96437956	0.27884902	h	1.00
-7.79030686	2.05580817	1.19733935	h	1.00
-9.70099086	0.54279654	-2.76977549	h	1.00
-5.05124568	-2.8535701	-6.04257413	h	1.00

4.23494183	2.24583584	-0.03603808	h	1.00
-6.97592793	4.46674984	-4.66309334	h	1.00
6.92220429	-1.05319927	-1.83764829	h	1.00
4.82710134	-4.97070813	1.88234275	h	1.00
7.51487268	0.04807471	2.77529407	h	1.00
2.04931983	-1.48858349	3.80434813	h	1.00
1.94516395	2.6885745	5.07732157	h	1.00
9.20352365	-4.84902687	2.93908496	h	1.00
6.45757433	-1.09003127	6.90766876	h	1.00
7.18737103	-5.41387832	-3.51437444	h	1.00
3.96383876	-5.93359804	-2.72681382	h	1.00
3.26005100	-2.35713988	-5.38892925	h	1.00

1a-2

Coordinates (Angstroms)			atom	charge
X	Y	Z		
1.30799867	5.16765154	1.92328358	c	6.00
-0.67349093	3.47086033	1.47761026	c	6.00
-3.12236183	4.3022431	0.74594256	c	6.00
-3.57592633	6.90552982	0.4612622	c	6.00
-1.60869881	8.59149199	0.8907515	c	6.00
0.80590999	7.72949804	1.61107483	c	6.00
-4.67634199	2.07531481	0.44182531	c	6.00
-3.16166546	0.05557319	1.00699591	c	6.00
-0.74106342	0.83760466	1.65922114	n	7.00
-7.25829975	1.88692317	-0.71785911	c	6.00
-7.1510074	2.18361696	-3.65846942	c	6.00
-4.95281673	0.59322966	-4.62312038	c	6.00
-5.335501	-1.90153055	-4.43076357	o	8.00

-2.97085498	1.52170028	-5.3521198	o	8.00
1.38769317	-0.8543631	1.39957117	c	6.00
-6.83569013	4.70948873	-4.41673501	o	8.00
3.19935875	-0.25509353	3.27939474	o	8.00
5.31347499	-1.96697346	3.35514204	c	6.00
6.7353818	-1.87890616	0.83539919	c	6.00
4.9063968	-2.4813118	-1.3114788	c	6.00
2.6231007	-0.71854527	-1.25190343	c	6.00
0.94654231	-1.52788081	-3.19408268	o	8.00
8.73330382	-3.65754892	0.93361955	o	8.00
6.25948837	-2.28089646	-3.61729936	o	8.00
6.89447462	-1.1577134	5.6218019	c	6.00
5.48744585	-1.28621725	7.88978919	o	8.00
3.15082229	4.49920945	2.5141561	h	1.00
-5.42768995	7.57422647	-0.11701003	h	1.00
-1.93354392	10.60704355	0.67422782	h	1.00
2.30546144	9.0919843	1.94239982	h	1.00
-3.60188636	-1.93914466	0.95509432	h	1.00

-8.10458352	0.05109271	-0.2718036	h	1.00
-8.54568118	3.35520824	-0.03254758	h	1.00
-8.92559604	1.48523718	-4.4697232	h	1.00
-3.73831245	-2.73626682	-4.80950362	h	1.00
0.65904091	-2.78410603	1.68085227	h	1.00
-5.03071386	4.99935773	-4.56905447	h	1.00
4.61555396	-3.90153275	3.67860955	h	1.00
7.46796166	0.05194417	0.54547192	h	1.00
4.2227589	-4.43525276	-1.04635071	h	1.00
3.25589083	1.22912149	-1.58219456	h	1.00
0.0199258	-0.09237467	-3.87628807	h	1.00
9.37541917	-3.80238897	-0.77373302	h	1.00
5.02811447	-2.50409322	-4.95253316	h	1.00
8.5054178	-2.43632529	5.80688134	h	1.00
7.63117443	0.76128326	5.27382729	h	1.00
3.96029099	-0.3223745	7.5970061	h	1.00

1a-3

Coordinates (Angstroms)			atom	charge
X	Y	Z		
1.17309115	6.43871799	0.52980542	c	6.00
-0.33109969	4.26636583	0.34312795	c	6.00
-3.01581264	4.39402994	0.23632949	c	6.00
-4.19596627	6.77723618	0.28646124	c	6.00
-2.70718192	8.93394471	0.44986009	c	6.00
-0.0518887	8.76419068	0.57272621	c	6.00
-3.94585839	1.82797094	0.11648503	c	6.00
-1.85794543	0.29035178	0.14701428	c	6.00
0.33767086	1.72641662	0.25875371	n	7.00
-6.64750735	0.96899262	-0.04258896	c	6.00
-7.85246987	1.27761962	-2.69817612	c	6.00
-6.4607166	-0.43210269	-4.56688197	c	6.00
-5.31675833	0.87281041	-6.44054708	o	8.00

-6.33384859	-2.70959609	-4.36797698	o	8.00
2.86671794	0.71115494	0.45904322	c	6.00
-8.03608845	3.81713954	-3.48465389	o	8.00
2.99283616	-1.44650553	-1.11982488	o	8.00
5.46136458	-2.59124013	-1.23222146	c	6.00
6.24208196	-3.49409831	1.40508323	c	6.00
6.14387815	-1.27380229	3.24514617	c	6.00
3.54699212	-0.02580309	3.19890579	c	6.00
3.69884438	2.12373356	4.799236	o	8.00
8.71319361	-4.50667221	1.24806854	o	8.00
6.76551539	-2.21666149	5.67769199	o	8.00
5.24858451	-4.70562149	-3.17602303	c	6.00
4.53047385	-3.75904432	-5.57016654	o	8.00
3.21599013	6.32700054	0.66964034	h	1.00
-6.23969051	6.91491809	0.16807021	h	1.00
-3.59897464	10.78244844	0.49043894	h	1.00
1.06453167	10.48104197	0.71210112	h	1.00
-1.7399817	-1.74489501	0.04426855	h	1.00

-6.78568011	-1.02744008	0.48168978	h	1.00
-7.82641184	2.04465899	1.27778793	h	1.00
-9.79381628	0.56098801	-2.60139219	h	1.00
-4.44499115	-0.36926349	-7.47711251	h	1.00
4.19113725	2.17903685	-0.18542959	h	1.00
-6.36657696	4.38480974	-3.96885351	h	1.00
6.83798103	-1.1857431	-1.9109421	h	1.00
4.8808944	-4.93556437	2.0504889	h	1.00
7.55173998	0.13788454	2.63433337	h	1.00
2.12579396	-1.38230313	3.88127118	h	1.00
2.0234254	2.82545539	5.00098469	h	1.00
9.27957203	-4.7468147	2.97151955	h	1.00
6.61489503	-0.81462001	6.84225119	h	1.00
7.09147724	-5.61371135	-3.38424933	h	1.00
3.90091236	-6.13054235	-2.47103252	h	1.00
3.01993483	-2.76759813	-5.28740312	h	1.00

1a-4

Coordinates (Angstroms)			atom	charge
X	Y	Z		
1.50550861	6.6226083	-0.37727139	c	6.00
0.21684312	4.56533103	-1.42819261	c	6.00
-2.19224587	4.84172264	-2.58539626	c	6.00
-3.32056877	7.24605045	-2.66650358	c	6.00
-2.04788347	9.28888726	-1.61914753	c	6.00
0.3436637	8.97732355	-0.48809462	c	6.00
-2.95816535	2.37029702	-3.45350487	c	6.00
-1.03874157	0.74652307	-2.82886797	c	6.00
0.88945789	2.02842032	-1.59038611	n	7.00
-5.50354421	1.6238576	-4.46930673	c	6.00
-6.86827529	-0.24922593	-2.66469376	c	6.00
-6.69291412	0.7007974	0.04730287	c	6.00
-7.97841233	2.83744507	0.44742552	o	8.00

-5.47055825	-0.37711825	1.68036964	o	8.00
2.87725567	0.81675994	-0.18505338	c	6.00
-5.75795334	-2.66644264	-2.81470926	o	8.00
4.65443182	-0.2433065	-1.9038469	o	8.00
6.86227928	-1.26029872	-0.68357668	c	6.00
6.09867712	-3.41257064	1.09767623	c	6.00
4.11624593	-2.46180572	2.96555032	c	6.00
1.87728502	-1.29148944	1.57419769	c	6.00
0.18278411	-0.37688905	3.43280152	o	8.00
8.29169714	-4.27399314	2.36940534	o	8.00
3.36111922	-4.53987293	4.48194556	o	8.00
8.6257406	-2.07620286	-2.80913125	c	6.00
9.27365782	-0.01408716	-4.38204448	o	8.00
3.35611351	6.41374139	0.483245	h	1.00
-5.16015182	7.50579476	-3.54206041	h	1.00
-2.89752503	11.15692031	-1.677027	h	1.00
1.30531258	10.60811601	0.30521912	h	1.00
-0.89990319	-1.25812702	-3.17528021	h	1.00

-6.69480051	3.29502977	-4.72485441	h	1.00
-5.37912842	0.67137402	-6.30352045	h	1.00
-8.87708936	-0.35783456	-3.19113876	h	1.00
-7.66010207	3.36147735	2.18081683	h	1.00
3.78419164	2.27190298	0.98511773	h	1.00
-5.18088217	-3.07743382	-1.12214356	h	1.00
7.7920602	0.23881973	0.42271689	h	1.00
5.24147225	-4.94273772	-0.03053472	h	1.00
4.99178741	-0.98491026	4.15135979	h	1.00
0.9974106	-2.76671457	0.40583604	h	1.00
-1.50809856	-0.26832088	2.73054046	h	1.00
7.7095587	-5.39331528	3.69484398	h	1.00
1.97846811	-3.93449927	5.51788848	h	1.00
10.37119679	-2.81727116	-1.99145118	h	1.00
7.71179349	-3.61102176	-3.88342048	h	1.00
7.69027864	0.75418608	-4.88204357	h	1.00

1a-5

Coordinates (Angstroms)			atom	charge
X	Y	Z		
0.95866125	6.26614655	0.89317918	c	6.00
0.05725185	4.32758545	-0.6724005	c	6.00
-2.22722246	4.56781529	-2.06979465	c	6.00
-3.61302752	6.83046831	-1.90194354	c	6.00
-2.70887583	8.76487296	-0.37421581	c	6.00
-0.4486377	8.4833909	1.00811866	c	6.00
-2.58526024	2.23636132	-3.4450268	c	6.00
-0.58121966	0.70384828	-2.8268039	c	6.00
1.04403285	1.94562126	-1.17994117	n	7.00
-4.7271533	1.56712864	-5.18546487	c	6.00
-6.91310868	0.1453881	-3.830767	c	6.00
-8.24715018	1.94497582	-2.00560813	c	6.00
-7.8728754	1.3397535	0.44609661	o	8.00
-9.45519682	3.77478062	-2.67251491	o	8.00

3.29417736	0.85383214	-0.07244191	c	6.00
-6.0995781	-2.12341879	-2.70489854	o	8.00
4.41053179	-0.72179476	-1.92339602	o	8.00
6.76077341	-1.8558424	-1.14162355	c	6.00
6.30817525	-3.54596742	1.16433836	c	6.00
5.09526297	-1.98821501	3.26769438	c	6.00
2.69706868	-0.69699334	2.32651542	c	6.00
1.82762353	0.8617333	4.33104592	o	8.00
8.66067444	-4.55039539	1.94597982	o	8.00
4.60722165	-3.64806942	5.31717576	o	8.00
7.75195525	-3.26626646	-3.44754454	c	6.00
8.18083509	-1.61065755	-5.5007231	o	8.00
2.67027639	6.04985354	2.00157187	h	1.00
-5.37980873	7.04395462	-2.92396408	h	1.00
-3.76314496	10.51994488	-0.22888973	h	1.00
0.20586167	10.02350257	2.19696111	h	1.00
-0.09774483	-1.15432287	-3.51878775	h	1.00
-5.50038604	3.26392163	-6.08084103	h	1.00

-4.05528715	0.31893025	-6.69281332	h	1.00
-8.32257132	-0.37593805	-5.2528793	h	1.00
-8.71875417	2.64598825	1.42428359	h	1.00
4.56954118	2.41423076	0.44139055	h	1.00
-4.86040634	-1.67408021	-1.43480489	h	1.00
8.11048045	-0.35514157	-0.63804285	h	1.00
4.98405416	-5.06583483	0.63147673	h	1.00
6.44700094	-0.50762337	3.84022733	h	1.00
1.29358211	-2.14825667	1.82868699	h	1.00
0.19778351	1.55299267	3.87692133	h	1.00
8.37074044	-5.32737161	3.57744749	h	1.00
3.77289079	-2.66250969	6.61229167	h	1.00
9.55549729	-4.14967156	-2.96643204	h	1.00
6.40281935	-4.77745443	-3.93771882	h	1.00
6.62074167	-0.69029263	-5.75368593	h	1.00

1b-1

Coordinates (Angstroms)			atom	charge
X	Y	Z		
1.19331941	4.85045378	1.24540193	c	6.00
0.15556006	3.27096902	-0.60927466	c	6.00
-2.02288941	3.96823117	-2.02115765	c	6.00
-3.15673099	6.32829947	-1.56345457	c	6.00
-2.11335378	7.91016476	0.25272656	c	6.00
0.0369295	7.17646586	1.64308099	c	6.00
-2.57339297	1.91077585	-3.73128884	c	6.00
-0.77569854	0.09515552	-3.29607146	c	6.00
0.89846361	0.88695474	-1.4338128	n	7.00
-4.68238195	1.73009729	-5.62174669	c	6.00
-7.31745596	1.19093531	-4.44460124	c	6.00
-7.21253862	-1.16946748	-2.80322008	c	6.00
-6.78184793	-3.29494824	-4.13498012	o	8.00

-7.4436113	-1.13234618	-0.52069683	o	8.00
2.88405444	-0.65271908	-0.37684499	c	6.00
-8.21649809	3.23309649	-2.9965685	o	8.00
4.1449875	-1.86605967	-2.40896304	o	8.00
6.27995388	-3.37014761	-1.64073332	c	6.00
5.40116253	-5.49069584	0.12446181	c	6.00
3.99899818	-4.34858443	2.37228402	c	6.00
1.84747893	-2.65803913	1.4724661	c	6.00
3.11570389	-6.37910941	3.88756716	o	8.00
7.48822815	-4.31112758	-4.0795396	c	6.00
0.77466679	-1.53951952	3.66099794	o	8.00
7.55069527	-6.86688928	0.925712	o	8.00
8.28547124	-2.28198193	-5.62449925	o	8.00
2.8246917	4.28941023	2.35501181	h	1.00
-4.84878627	6.86584421	-2.58972327	h	1.00
-2.97444945	9.73615549	0.62520924	h	1.00
0.8019039	8.44450207	3.06494805	h	1.00
-0.48047205	-1.69722523	-4.22879195	h	1.00

-4.87307342	3.50010358	-6.68106333	h	1.00
-4.27060526	0.23602741	-6.99393947	h	1.00
-8.6537861	0.84004293	-5.99494314	h	1.00
-6.70956689	-4.67393522	-2.92200586	h	1.00
4.18035461	0.60197192	0.65369985	h	1.00
-7.98999264	2.76244145	-1.23651454	h	1.00
7.64405866	-2.16300756	-0.63413397	h	1.00
4.0727508	-6.71740457	-0.91449552	h	1.00
5.34328041	-3.17370451	3.44939416	h	1.00
0.45441216	-3.81997389	0.45470419	h	1.00
2.14939642	-5.63522716	5.25095323	h	1.00
9.15401704	-5.4383317	-3.61189387	h	1.00
6.12836731	-5.54713951	-5.06433252	h	1.00
-0.76489748	-0.67705455	3.18419304	h	1.00
6.98585056	-7.95590613	2.28373663	h	1.00
6.83537907	-1.18264005	-5.81248263	h	1.00

1b-2

Coordinates (Angstroms)			atom	charge
X	Y	Z		
0.78320217	4.53792469	0.79237012	c	6.00
-0.64127866	2.42115583	0.07796815	c	6.00
-3.2974799	2.55622489	-0.31859819	c	6.00
-4.53018844	4.89302698	-0.02255726	c	6.00
-3.11864109	6.9977837	0.66364315	c	6.00
-0.4920043	6.81931269	1.07012432	c	6.00
-4.15025806	0.04229465	-0.96132984	c	6.00
-2.0417152	-1.47285599	-0.94690772	c	6.00
0.09223787	-0.06698596	-0.34390955	n	7.00
-6.79011089	-0.78107193	-1.60763069	c	6.00
-7.37937334	-0.65072669	-4.48393736	c	6.00
-7.48671797	2.11290822	-5.32067279	c	6.00
-5.58680076	2.74710752	-6.89948072	o	8.00

-9.06425865	3.60568434	-4.58764937	o	8.00
2.60329351	-1.09414159	-0.00024036	c	6.00
-5.7274747	-2.15408233	-5.92699291	o	8.00
2.93419452	-3.02332294	-1.82535907	o	8.00
5.41553239	-4.14803425	-1.80921481	c	6.00
5.90984553	-5.39071833	0.75855219	c	6.00
5.5828289	-3.43000704	2.84871029	c	6.00
2.99107917	-2.18287013	2.67892097	c	6.00
5.94531592	-4.68276475	5.19211194	o	8.00
5.45562281	-5.99047874	-4.02096337	c	6.00
2.9404161	-0.26220479	4.55378316	o	8.00
8.39468888	-6.38095719	0.7475804	o	8.00
5.05189215	-4.74166987	-6.34865506	o	8.00
2.79798231	4.41082488	1.15260079	h	1.00
-6.54932172	5.05090092	-0.3547812	h	1.00
-4.05054915	8.81169847	0.89859897	h	1.00
0.56074689	8.49366164	1.61976035	h	1.00
-1.86683643	-3.46770565	-1.34161078	h	1.00

-7.07656431	-2.74906917	-1.03712519	h	1.00
-8.18427492	0.36823666	-0.59942191	h	1.00
-9.28057093	-1.4128667	-4.7839488	h	1.00
-5.76620907	4.54705249	-7.22527616	h	1.00
3.96556328	0.44911213	-0.29773187	h	1.00
-4.05534032	-1.42735342	-5.76609615	h	1.00
6.84031328	-2.66834423	-2.14023646	h	1.00
4.50269934	-6.90054888	1.0550887	h	1.00
7.03181936	-1.95350937	2.58496612	h	1.00
1.52156947	-3.61409843	3.01899638	h	1.00
5.64941434	-3.44457807	6.50527956	h	1.00
7.31220368	-6.89030002	-4.10667846	h	1.00
4.0331392	-7.47499019	-3.67965883	h	1.00
1.24455639	0.41042776	4.66325787	h	1.00
8.77052217	-6.84401103	2.47769736	h	1.00
3.49804608	-3.79627196	-6.16136099	h	1.00

1b-3

Coordinates (Angstroms)			atom	charge
X	Y	Z		
0.73811838	4.63355258	1.03885765	c	6.00
-0.67593516	2.53861805	0.24715236	c	6.00
-3.32297061	2.69113333	-0.20724805	c	6.00
-4.54738724	5.0290541	0.10590201	c	6.00
-3.14636694	7.11550838	0.8659547	c	6.00
-0.53112135	6.91671158	1.33177924	c	6.00
-4.16749833	0.19888485	-0.92682651	c	6.00
-2.07029932	-1.32622787	-0.89788574	c	6.00
0.05324894	0.05709095	-0.21140953	n	7.00
-6.79658587	-0.57374844	-1.67040085	c	6.00
-7.12030742	-1.03270804	-4.54440311	c	6.00
-6.38417118	1.28601793	-6.08144564	c	6.00
-7.7730571	3.34189624	-5.52732456	o	8.00

-4.7110059	1.26777321	-7.65156334	o	8.00
2.56098946	-0.97808087	0.11321851	c	6.00
-5.66435837	-3.1094631	-5.34605038	o	8.00
2.93706066	-2.79716695	-1.81332594	o	8.00
5.42430594	-3.90339392	-1.82113298	c	6.00
5.88544036	-5.29056955	0.67810957	c	6.00
5.50638019	-3.4571091	2.8732359	c	6.00
2.90884099	-2.22019748	2.72992225	c	6.00
5.83978062	-4.84153125	5.14674825	o	8.00
5.50969222	-5.61106506	-4.13776874	c	6.00
2.80697007	-0.4126076	4.71246097	o	8.00
8.37814182	-6.26205603	0.65523669	o	8.00
5.11856048	-4.22810492	-6.39011571	o	8.00
2.74400132	4.49051078	1.44159691	h	1.00
-6.56142005	5.20161425	-0.2542603	h	1.00
-4.07606357	8.928879	1.11663019	h	1.00
0.51564995	8.57441672	1.93995857	h	1.00
-1.8947339	-3.29753793	-1.3886931	h	1.00

-7.32887846	-2.34843423	-0.74607863	h	1.00
-8.16706638	0.85416744	-1.06718725	h	1.00
-9.13510453	-1.40361393	-4.91359855	h	1.00
-7.12109204	4.71764072	-6.55702194	h	1.00
3.92010298	0.58557478	-0.06889151	h	1.00
-4.47643215	-2.44712043	-6.58163089	h	1.00
6.84512603	-2.39856792	-2.04172761	h	1.00
4.48548234	-6.82496271	0.86088716	h	1.00
6.94808555	-1.95715061	2.72201801	h	1.00
1.44409384	-3.67879431	2.9582474	h	1.00
5.50502669	-3.68440328	6.52293344	h	1.00
7.375864	-6.48743212	-4.25524852	h	1.00
4.09789048	-7.12643605	-3.90214105	h	1.00
1.10726589	0.25200152	4.81094079	h	1.00
8.72503765	-6.8228132	2.36229633	h	1.00
3.56246804	-3.29573602	-6.15646228	h	1.00

1b-4

Coordinates (Angstroms)			atom	charge
X	Y	Z		
0.94025078	4.68221896	1.45940232	c	6.00
0.04680679	3.17789454	-0.52919857	c	6.00
-2.06844632	3.89904531	-2.02696685	c	6.00
-3.27549028	6.21426551	-1.51990801	c	6.00
-2.37639876	7.72070576	0.43366824	c	6.00
-0.29374854	6.95973089	1.90831791	c	6.00
-2.47765024	1.91617284	-3.86041448	c	6.00
-0.66420978	0.11782607	-3.41088746	c	6.00
0.88846006	0.85622922	-1.4267953	n	7.00
-4.47532842	1.80197326	-5.87472207	c	6.00
-7.13987416	1.09320779	-4.8753575	c	6.00
-7.05169907	-1.58095189	-3.78385129	c	6.00
-7.54256148	-1.64996657	-1.27554439	o	8.00

-6.5340361	-3.44792432	-5.00335271	o	8.00
2.92059711	-0.6445673	-0.38271153	c	6.00
-8.16003649	2.90520561	-3.20865533	o	8.00
4.11752714	-1.90093138	-2.4189641	o	8.00
6.27764648	-3.38878816	-1.68592881	c	6.00
5.45314343	-5.46786524	0.15301683	c	6.00
4.13495885	-4.26878338	2.42222341	c	6.00
1.95468786	-2.59386933	1.56192698	c	6.00
3.3063642	-6.26054483	4.01715711	o	8.00
7.41004361	-4.38375013	-4.13937616	c	6.00
0.98061802	-1.40631266	3.76376878	o	8.00
7.62626889	-6.83046321	0.91106262	o	8.00
8.18057419	-2.39030091	-5.74347214	o	8.00
2.51448767	4.09269588	2.63457578	h	1.00
-4.90453132	6.78880115	-2.62753622	h	1.00
-3.29944237	9.50750129	0.84476382	h	1.00
0.35912192	8.16824824	3.4338616	h	1.00
-0.28984518	-1.62797924	-4.40019932	h	1.00

-4.65834129	3.63518216	-6.82170227	h	1.00
-3.95030807	0.40838748	-7.31005901	h	1.00
-8.42800636	1.00992598	-6.49509447	h	1.00
-7.39888968	-3.41368263	-0.77948639	h	1.00
4.24105514	0.65041022	0.56875292	h	1.00
-7.26008748	2.7881005	-1.62150479	h	1.00
7.67159524	-2.15843087	-0.75088571	h	1.00
4.08676897	-6.71273694	-0.81216761	h	1.00
5.51988306	-3.07125324	3.42006742	h	1.00
0.51414277	-3.77438431	0.63536081	h	1.00
2.41536496	-5.48520903	5.41328812	h	1.00
9.08056155	-5.51310838	-3.6947992	h	1.00
6.01608729	-5.62855829	-5.06260495	h	1.00
-0.52197682	-0.46970429	3.30990225	h	1.00
7.10372031	-7.89823546	2.3023658	h	1.00
6.7257875	-1.29983899	-5.94315123	h	1.00
